# Long Non-Coding RNAs: Novel Players in Regulation of Immune Response Upon Herpesvirus Infection

**DOI:** 10.3389/fimmu.2018.00761

**Published:** 2018-04-12

**Authors:** Waqas Ahmed, Zheng-Fei Liu

**Affiliations:** ^1^State Key Laboratory of Agricultural Microbiology, College of Veterinary Medicine, Huazhong Agricultural University, Wuhan, China; ^2^College of Life Sciences, Guangzhou University, Guangzhou, China; ^3^Key Laboratory of Preventive Veterinary Medicine in Hubei Province, College of Veterinary Medicine, Huazhong Agricultural University, Wuhan, China

**Keywords:** herpesvirus, long non-coding RNAs, virus infection, innate immunity, adaptive immunity, host–pathogen interaction

## Abstract

Herpesviruses have developed a variety of sophisticated immune evasion strategies to establish lifelong latent infection, including the use of long non-coding RNAs (lncRNAs). In this review, we summarize the lncRNA action modes, i.e., RNA–protein, RNA–RNA, and RNA–DNA interactions, involved in regulating important aspects of immunity by controlling gene expression at various stages. Upon herpesvirus infection, host lncRNAs, such as nuclear paraspeckle assembly transcript 1, negative regulator of antiviral, and B-cell integration cluster have been functionally characterized as negative or positive antiviral regulators in the immune response. Herpesviruses have also evolved multiple strategies to modulate the host immune response using lncRNAs, such as latency-associated transcript, β 2.7 RNA, 5 kb and 7.2 kb lncRNAs, Epstein–Barr virus-encoded non-coding RNA, *Bam*H I-A rightward transcripts, polyadenylated nuclear, and herpesvirus saimiri U-rich RNAs. We discuss the various mechanisms of immune-related lncRNAs, and their diversified and important functions in the modulation of innate and adaptive immunity upon herpesvirus infection as well as in host–pathogen interactions, which will facilitate our understanding of rational design of novel strategies to combat herpesvirus infection.

## Introduction

**Figure 1:**
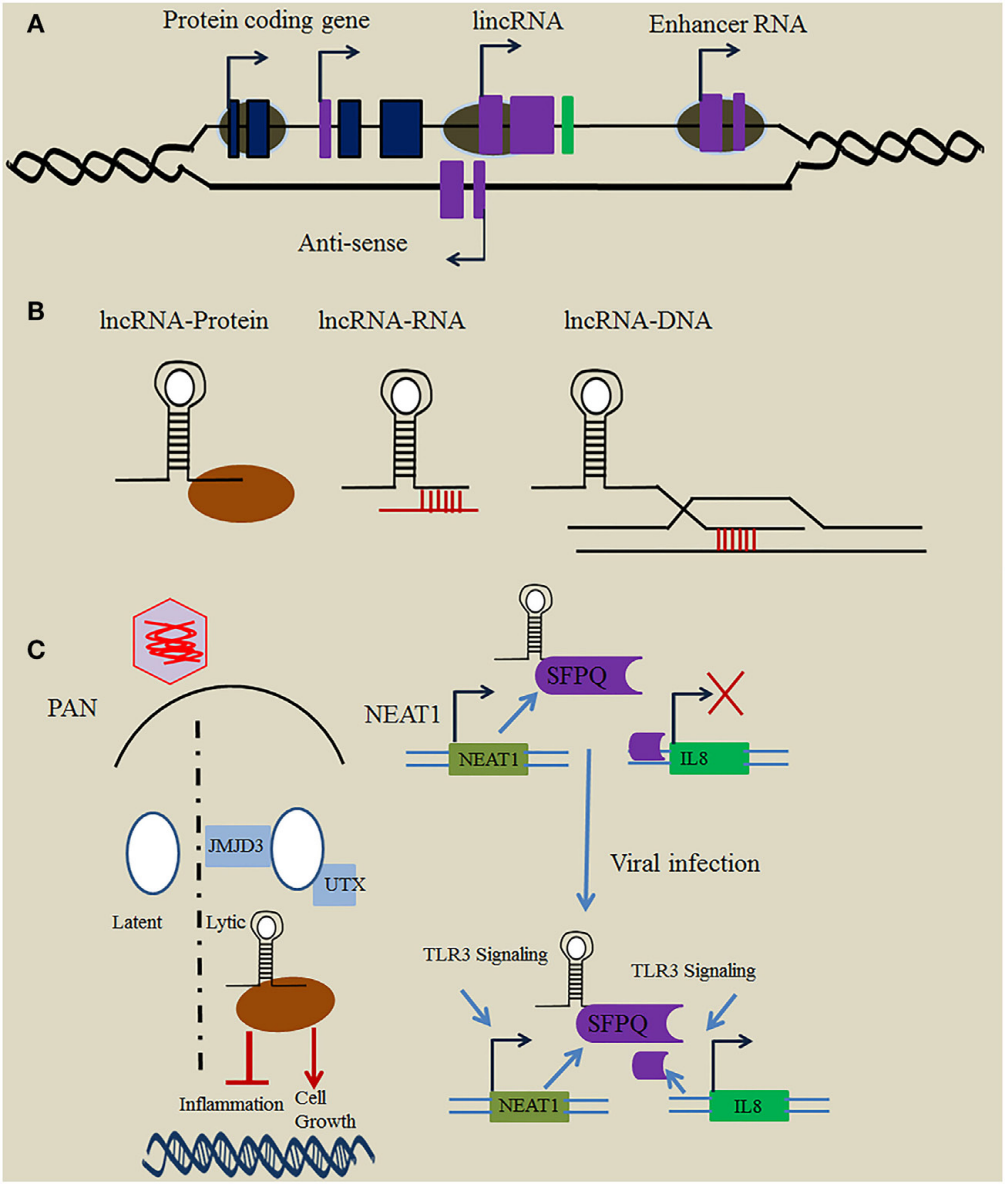
Mechanism employed by long non-coding RNA (lncRNA) in immune regulation. **(A)** Loci of immune-related lncRNAs relative to protein-coding genes. In general, immune-related lncRNAs are transcribed by RNAP II and classified relative to the position of the neighboring protein-coding gene into different types, including intronic lncRNA, long intergenic ncRNA (lincRNA), enhancer RNA (eRNA), and antisense lncRNA. **(B)** Mechanisms employed by lncRNAs. lncRNAs use a variety of basic modules to perform their regulatory functions in the cytosol and nucleus through lncRNA–protein, lncRNA–RNA, and lncRNA–DNA interactions. **(C)** Function of herpesviruses’ lncRNAs in immune regulation. The Kaposi’s sarcoma-associated herpesvirus (KSHV)-encoded lncRNA polyadenylated nuclear (PAN) binds the histone-modifying complex (demethylases JMJD3 and UTX) which plays an important role in the switch from latent to lytic infection. PAN also subverts the host immune response and modulates viral gene expression through binding with PRC2 to promote cell growth and survival, and to repress the inflammatory response. Nuclear paraspeckle assembly transcript 1 (NEAT1) binds several proteins such as SFPQ, and plays a critical role in regulation of the innate immune response mechanism through the transcriptional regulation of numerous antiviral genes upon herpesvirus infection.

Non-coding RNAs (ncRNAs) play critical roles in cellular functions and are arbitrarily classified as either short ncRNAs (<200 nucleotides) or long ncRNAs (>200 nucleotides) (1–3). Long non-coding RNAs (lncRNAs) are transcribed by RNA polymerase II and do not encode information about proteins. Based on their position relative to protein-coding genes, they are broadly divided into different classes, such as intronic lncRNAs, long intergenic ncRNAs (lincRNAs), antisense lncRNAs, bidirectional lncRNAs, transcribed pseudogene lncRNAs, and enhancer RNAs (eRNAs) (Figure 1A) (4). Thousands of lncRNAs are encoded within the human genome and have been assumed to be “junk” or “dark matter” (5).

The function of lncRNAs has been investigated in a wide range of species, including plants, animals, prokaryotes, and viruses. They were shown to be poorly conserved among species, invoking uncertainty about functional species-specific characteristics (6). Growing evidence suggests that lncRNAs contribute to a wide range of biological functions, including cellular structure integrity, splicing, transcription, translation, stem cell pluripotency, the cell cycle, reprogramming, and apoptosis (7–9). lncRNAs were also found to be involved in the development of many human diseases and may regulate cancer progression (10, 11). Hence, biological features and functions of lncRNAs make them as an important and interesting research topic.

Herpesviruses are large, enveloped, double-stranded DNA viruses that have infected a wide range of species including animals and humans for hundreds of millions of years. They are classified into three subfamilies: *Alphaherpesvirinae, Betaherpesvirinae*, and *Gammaherpesvirinae* (12, 13). These viruses can establish lifelong infections in their hosts and are extensive within the human population (14). In immune-compromised individuals, herpesvirus infections result in substantial disease symptoms with a wide range of clinical manifestations spanning from congenital defects, skin/mucosal lesions, and cancer development (15, 16).

The ability to establish latency is a major hallmark of herpesvirus infection, and herpesviruses have evolved numerous strategies to evade immune response mechanism to avoid recognition during establishing the latent infection. Recent emerging evidence demonstrates that lncRNAs play key regulatory roles in pathological and physiological responses (17, 18). lncRNAs potential function in regulation of immune response mechanism upon herpesvirus infection is now growing and actually the subject that signifies the topic of this review. lncRNAs associated with immune-related functions are usually recognized through analysis of differential expression in response to activation of immune cells. The role of specific lncRNAs will be studied under different sections, such as innate immunity, adaptive immunity, and host–pathogen interaction. Nonetheless, understanding the mechanism of action of immune-related lncRNAs will undoubtedly shed more light on their function in immune regulation upon herpesvirus infection.

## Mechanism of Action and Function of Immune-Related lncRNAs

RNA-sequencing analysis revealed that most immune-related lncRNAs partially overlap or are close to the 3′ end (downstream) or 5′ end (upstream) of protein-coding genes and play key roles in the regulation of immune response mechanisms (19, 20). The transcription of immune-related lncRNAs usually occurs in the antisense direction, indicating that upstream lncRNAs and mRNAs use a common promoter region to produce bidirectional transcription. Surprisingly, antisense lncRNAs often have no or only a partial overlap with protein-coding genes (21). Many immune-related lncRNAs, including PACER (22), lnc-IL7R (23), THRIL (24), and lincR-Ccr2-50AS (19), have been exposed to have gene regulatory functions in their nearby protein-coding gene in *cis*.

Although chromatin looping is thought to be essential for placing distal enhancer regions in close proximity with promoter regions, eRNAs have also been reported to show gene regulatory functions in *cis*. lncRNAs located in intergenic regions, such as HOTAIR, control gene expression to modulate the immune response mechanism in *trans* (25). Similar to proteins, lncRNAs have modular domains that either enable protein binding through higher order structures or secondary RNA structures by base pairing (Figure 1B) (1, 26). Furthermore, lncRNAs also play critical roles as guides, scaffolds, decoys, and signals to regulate a variety of biological processes, including posttranscriptional regulation, transcription, and chromatin remodeling (27). Tumor necrosis factor (TNF) induces the expression of hundreds of lncRNAs in murine fibroblasts. Lethe, a pseudogene lncRNA, is transcribed upon activation of NF-κB, a transcription factor important in inflammation. Lethe binds directly with RelA–RelA homodimers that influence NF-κB response elements, thus hampering the function of downstream effectors, such as NF-κB, interleukin (IL)-6, and IL-8 (28). These findings suggest that Lethe functions as a post-induction feedback regulator of TNF signaling to dampen the inflammatory response. The muscle-specific lncRNA linc-MD1 is expressed during myoblast differentiation, and governs ceRNA to regulate the distribution of miR-135 and miR-133, in turn activating muscle-specific genes *MAML1* and *MEF2C* and regulating myoblast differentiation (29).

## lncRNAs in Modulation of Innate and Adaptive Immune Response upon Herpesvirus Infection

Host innate and adaptive immune response mechanisms are comprised of complexes of different biochemical processes regulated by lipid mediators and various proteins, such as pattern recognition receptors, chemokines, cytokines, prostaglandins, hormones, and growth factors. Most lncRNAs were initially thought to be involved in cell differentiation, cancer, and genomic imprinting (30), but growing evidence highlights their function in regulation of both the innate and adaptive immune systems (31, 32). Indeed, lncRNAs regulate dendritic cells, macrophages, activator of toll-like receptor 4 (TLR4) signaling, type I interferon (IFN) signaling, T-cell development, and differentiation (33).

Herpesviruses have established well-organized strategies that allow them to exploit and/or evade host immune response mechanisms to persist within its natural host. The function of lncRNAs in immune homeostasis to establish and maintain a herpesvirus latent infection is intriguing, but their biological function in immune regulation is only beginning to emerge. In the following sections, we describe immune-related lncRNAs that play critical roles in modulating the immune response mechanism of herpesviruses through unique mechanisms.

## Host lncRNAs

Host lncRNAs have been functionally characterized as negative or positive antiviral regulators in the immune response. Interestingly, a number of host lncRNAs can be hijacked and induced by viruses to develop persistent infections, which likely reflects the mutual adaptability of hosts and viruses that has evolved over millions of years. Many non-coding, antisense transcripts have been identified following infection with herpes simplex virus (HSV)-1, with the induction of antisense transcription providing protection against apoptosis. Natural antisense transcripts are involved in regulating gene expression during an immune challenge, suggesting that herpesviruses induce widespread host antisense transcription to interfere with the expression of pro-apoptotic genes (34). Pathogen invasion triggers a number of cellular responses and alters the host transcriptome, as shown for HSV-1 infection which leads to differential gene expression, and changes in RNA splicing and RNA Pol II read-through (35).

## Nuclear Paraspeckle Assembly Transcript 1 (NEAT1)

Nuclear paraspeckle assembly transcript 1 plays an important role in the establishment of nuclear paraspeckles, which are architectural subnuclear component structures formed during the nucleocytoplasmic transport of mRNA in response to stress stimuli (36). It is prompted by HSV infection which leads to the formation of large paraspeckles. NEAT1, also known as virus-inducible ncRNA, binds with several proteins present in paraspeckles such as splicing factor proline and glutamine rich (SFPQ) (37). Imamura et al. (37) explored the function of NEAT1 in the antiviral response within A549 and HeLa cell lines, and demonstrated that NEAT1 accelerates the expression of various antiviral genes such as IL-8. NEAT1 overexpression and knockdown led to the recognition of 85 genes that are directly controlled by NEAT1, including those that play key roles in the antiviral response. SFPQ-NEAT1 binding at the IL-8 promoter site represses IL-8 transcription; however, higher NEAT1 expression induces the formation of paraspeckles, resulting in transcriptional activation of IL8. Furthermore, hexamethylene bis-acetamide-inducible protein 1 (HEXIM1) binding with NEAT1 forms a multi-subunit complex that regulates the innate immune response through a cGAS-STING-IRF3-dependent pathway. The HEXIM1-DNA-PK-paraspeckle components-ribonucleoprotein complex contains various paraspeckle proteins, including MATRIN3, SFPQ, PSPC1, RBM14, and NONO. NEAT1 binding to HEXIM1 was shown to be essential for the formation of this complex (38).

**Figure 2:**
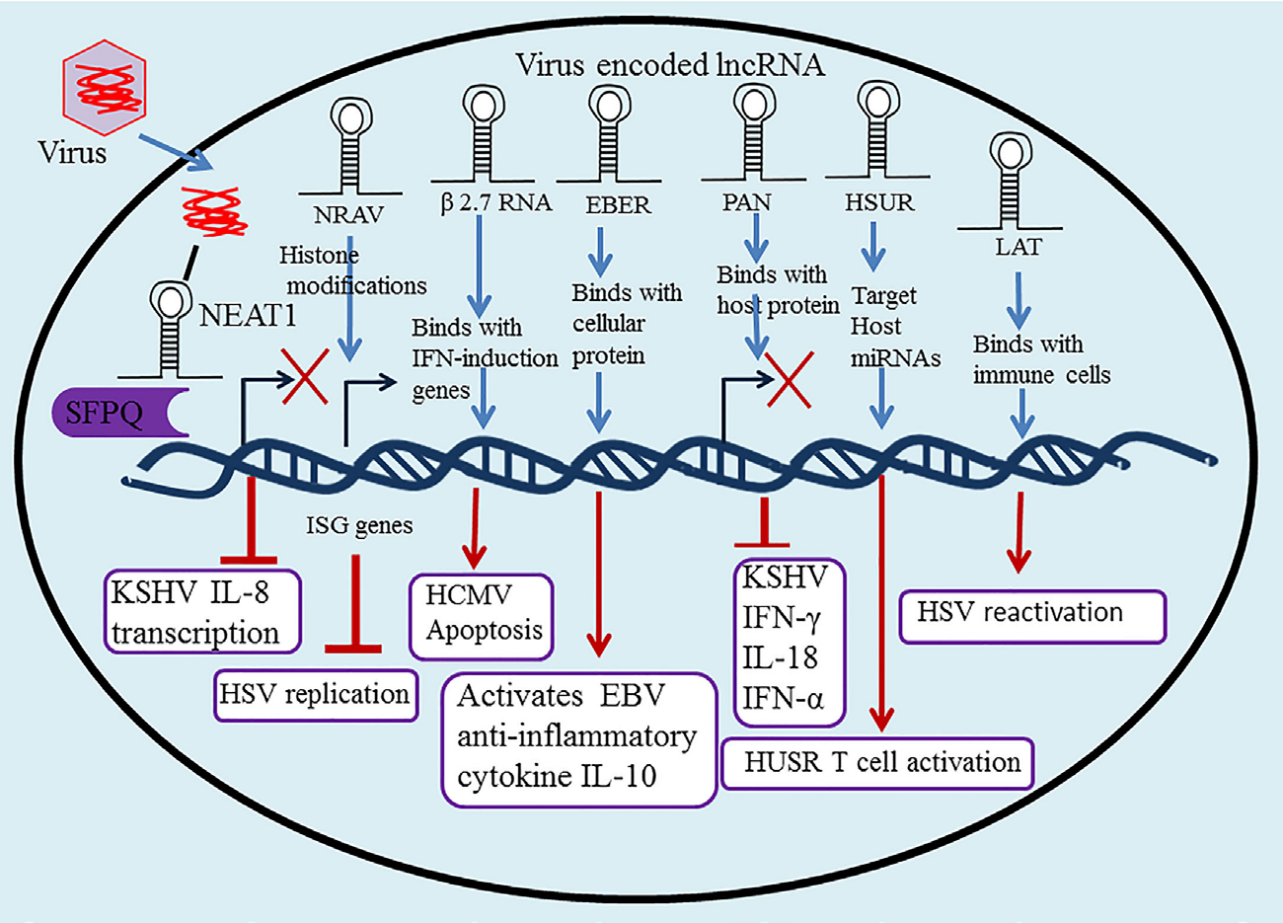
Long non-coding RNA (lncRNA) in regulation of the innate and adaptive immune system upon herpesvirus infection. Many lncRNAs have been associated with regulation of the innate and adaptive immune response and host–pathogen interactions, including nuclear paraspeckle assembly transcript 1 (NEAT1), negative regulator of antiviral (NRAV), polyadenylated nuclear (PAN), Epstein–Barr virus-encoded non-coding RNAs (EBERs), herpesvirus saimiri U-rich RNAs (HSURs), β 2.7 RNA, and latency-associated transcript (LAT).

Nuclear paraspeckle assembly transcript 1 upregulated gene expression was observed in Kaposi’s sarcoma-associated herpesvirus (KSHV) infection, indicating that KSHV benefits from NEAT1 targeting by preventing the death of KSHV-infected cells (39). In addition, HSV-1 infection leads to NEAT1 upregulated gene expression, resulting in STAT3-dependent paraspeckle formation (40). NONO is a multifunctional DNA-binding protein that is involved in transcriptional regulation of Epstein–Barr virus (EBV). Cao and colleagues reported novel *oriP*-derived leftward lncRNAs that plays critical function in facilitating viral lytic gene expression, undoubtedly in a manner that initiates the lytic cascade of herpesviruses (41). Taken together, NEAT1 regulates the innate immune response mechanism upon herpesvirus infection by NEAT1 and SFPQ cooperative mechanisms through the transcriptional regulation of numerous antiviral genes (Figure 2).

## Negative Regulator of Antiviral (NRAV)

Negative regulator of antiviral is a newly identified lncRNA with an essential function in the regulation of antiviral innate immunity. The downregulation of lncRNA NRAV is assumed to be linked with HSV infection. NRAV facilitates virus replication and significantly downregulates the expression of IFN-stimulated genes (ISGs), including *OASL, IFIT2, MxA, IFIT3*, and *IFITM3* (42). A chief mechanism of lncRNA function is the modular pairing of DNA binding and protein interaction to recruit chromatin-modifying proteins that regulate gene regulation *via* the chemical modification of histones. NRAV also regulates histone modifications H3K27me3 and H3K4me3 of ISG genes, which directly inhibits the initial transcription of *IFITM3* and *MxA* (42). ZO-1-associated nucleic acid binding protein is an NRAV-bound protein that acts as a positive regulator; these proteins interact to repress ISG expression in uninfected cells. Taken together, these results indicate that decreases in NRAV might increase the host innate immune defense through the involvement of various antiviral proteins (such as ISGs), thus enabling more efficient viral clearance. NRAV provides good evidence that lncRNAs regulate the antiviral IFN response.

## B-Cell Integration Cluster

The BIC was identified as a non-coding RNA linked with the regulation of numerous aspects of the immune system (43). It is an essential precursor of miR-155, which is highly expressed in activated cells of the immune system (44). BIC is induced by TNF-α, IFN-β, IFN-γ, TLR signaling, B-cell receptor, and EBV LMP1 and LMP2A engagement (45). Interestingly, the viral miRNA miR-K11, encoded by KSHV, is a functional ortholog of miR-155. EBV was recently shown to diminish NF-κB signaling and suppress host innate immunity to stabilize latent virus persistence by inducing miR-155 translational repression (46). Furthermore, BIC has critical functions in lymphomagenesis through the activation of p38/MAPK and NF-κB signaling pathways in response to EBV LMP1 (47).

## Viral lncRNAs

During viral infections, the host cell responds by producing numerous lncRNAs. Herpesviruses not only alter the expression of immune-related host lncRNAs but also generates their own lncRNAs to modulate the host immune response. In the following section, we describe herpesvirus-encoded lncRNAs that have been identified thus far.

## Latency-Associated Transcript (LAT)

Latency-associated transcript, a non-protein coding RNA that has been strongly implicated in the epigenetic regulation of HSV-1 gene expression, a phenomenon that directly impacts upon the frequency of reactivation and the maintenance of the transcriptionally active latent reservoir (48). LAT constitute a novel immune evasion mechanism whereby the HSV-1 LAT directly or indirectly promotes functional exhaustion (i.e., dysfunction) of HSV-specific CD8+ T cells in latently infected TG, resulting in increased virus reactivation (49). Furthermore, LAT increases reactivation by a direct effect on the reactivation process or by increasing the establishment of latency, thereby making more latently infected neurons available for reactivation (50). HSV-1 LAT contribute to the shaping of a broader repertoire of exhausted HSV-specific CD8+ T cells in latently infected TG *via* producing less TNF alpha, gamma IFN, and granzyme B, higher levels of PD-1, TIM-3, and CTLA-4 markers of exhaustion, and recognized a broader selection of nonoverlapping HSV-1 epitopes, thus allowing for increased viral reactivation (51).

## β 2.7 RNA

The 2.7-kb unspliced polyadenylated lncRNA β 2.7 RNA is the most abundant RNA transcribed in the human cytomegalovirus (HCMV) genome, accounting for some 20% of all viral RNAs transcript expressed at the early phase of lytic infection (52). Although it possesses coding potential, β 2.7 RNA directly binds to genes associated with retinoid/IFN-induced mortality 19, a subunit of the mitochondrial enzyme complex I. This complex stabilizes the mitochondrial membrane potential which protects virus-infected cells from apoptosis, resulting in continued adenosine triphosphate production for the successful completion of the viral life cycle (53). β 2.7 RNA interacts with complex I to prevent rotenone stress-induced apoptosis during both lytic and latent infection in neuronal cells, suggesting a refined strategy by which the virus regulates the metabolic viability of the host cell. Thus, by targeting complex I, β 2.7 RNA can be used in the development of a novel therapeutic tool (54). β 2.7 RNA is also a novel effector molecule that reduces the formation of reactive oxygen species by inducing apoptosis, and provides protection to rat aortic endothelial cells from reperfusion/ischemia injury (55).

## 5 and 7.2 kb lncRNAs

Human cytomegalovirus is a member of *Betaherpesvirinae* that persistently replicates in glandular epithelial tissue and ultimately develops a lifelong latent infection in the host. HCMV expresses a 5-kb immediate-early stable intronic lncRNA that lacks open reading frames and is highly AT-rich in sequence. It is likely to be translated into protein, so is not required for proliferation of the virus in HCMV-infected cultured fibroblasts (56). A 7.2-kb lncRNA expressed by murine CMV was shown to be an essential element of viral persistence in the salivary gland, facilitating progression from the acute to latent phase of infection (57, 58). The CMV intronic lncRNA is extremely long-lived and accumulates in the nuclei of cells during infection, suggesting that stability is a consequence of a sustained lariat conformation (59, 60).

## EBV-Encoded Non-Coding RNAs (EBERs)

EBV-encoded non-coding RNAs EBER1 (167 nt) and EBER2 (172 nt) are two abundant, highly structured, nuclear RNA transcripts produced during latent infection (61). They are nonpolyadenylated, ncRNA transcripts usually transcribed by RNA polymerase III, and involved in regulation of the antiviral innate immune response mechanism through direct interaction with cellular proteins (62). EBERs are recognized by RIG-I, and induce the expression of anti-inflammatory cytokine IL-10 to activate downstream signaling pathways but not NF-κB activation in EBV-infected cells (63, 64). They also play important roles in oncogenesis. For example, they counteract IFN-α-induced apoptosis through binding with (ds) RNA-activated protein kinase, resulting in inhibition of its phosphorylation (65, 66). EBERs can be identified by their expression of TLR3, which induces inflammatory cytokines and type-I IFNs. Because they are secreted from EBV-infected cells, they can be collected from the sera, suggesting subsequent immune activation using a TLR3-dependent signaling pathway (67).

## *Bam*H I-A Rightward Transcripts

*Bam*H I-A rightward transcripts are non-coding regulatory RNAs that perform identical functions to cellular lncRNAs in the regulation of cellular and/or viral gene expression. Nuclear BART RNAs show increased gene expression which contributes to growth regulation and viral oncogenesis without the expression of immunogenic proteins in EBV-infected cells. This elaborates the generalized mechanism of EBV epithelial latency in the presence of a functional immune system (68). NF-κB prompts the activation of BART promoters and controls the expression of BARTs in EBV-infected cells, as shown by the abolition of BART promoter activity through the introduction of mutations into putative NF-κB binding sites (69, 70).

## Polyadenylated Nuclear

The novel abundant 1.2-kb PAN RNA was first investigated in KSHV where it is transcribed by RNA Poly II. PAN RNA plays an important function in controlling viral gene expression and propagation and is involved in subversion of the host immune response (71, 72). PAN RNA typically influences cellular and viral gene expression *via* binding with host proteins, including histone methyltransferase MLL2, histones H1 and H2A, the demethylase UTX, poly (A)-binding protein C1, IFN regulatory factor 4, and polycomb repression complex 2 proteins SUZ12, EZH2, and JMJD3 (73, 74). Its interactions with histone-modifying complexes eliminate the suppressive H3K23me3 mark on the KSHV genome, thus activating lytic replication (Figure 1C). This activation leads to decreased production of viral and inflammatory genes, increased survival, and an enhanced growth phenotype through the epigenetic regulation of KSHV gene expression (75).

Polyadenylated nuclear RNA also decreases the expression of inflammatory cytokines, such as IFN-α, IFN-γ, and IL-18, suggesting it functions as an immune modulator (76, 77), while its interaction with the latency-associated nuclear antigen shows it has a role in the maintenance of KSHV latency. The binding of PAN RNA with the viral genome contributes to the switch from latent to lytic infection (78, 79). Taken together, these studies demonstrate that viral lncRNA PAN functions as a major global regulator in the regulation of host as well as viral gene expression.

## Herpesvirus Saimiri U-Rich RNAs (HSURs)

Herpesvirus saimiri U-rich RNAs are viral lncRNAs functioning as miRNA sponges, which cause aggressive lymphoma and T-cell leukemia in latently herpesvirus saimiri (HVS)-infected marmoset T cells (80). Cazalla et al. (81) documented sequence complementarity between HSUR1 and HSUR2, revealing that target host miRNAs are differently expressed in T cells. They also showed that HSUR1 and 2 significantly downregulate miR-16 and miR-27, clarifying a viral lncRNA strategy that modulates host-cell gene expression through manipulating the miRNA pathway in a binding dependent, sequence-specific manner (81). A recent study examined the role of miRNAs in HVS-transformed T cells, showing that host miRNAs are directly involved in the downregulation of numerous proteins in the T cell receptor signaling pathway (80). HSURs 1 and 2 were also reported to upregulate the expression of host genes linked to T cell activation in HVS-infected cells (82). This relationship between host miRNAs and virally encoded lncRNAs may prove to be an important function of viral pathogenesis (83).

## Conclusion and Future Perspectives

Long non-coding RNAs are fundamental constituents of cells that are considered a major class of RNA genes encoded by the genome, and transcribed and spliced into functional ncRNA molecules. They have been shown to play essential roles in the expression of protein-coding genes, genomic imprinting, genomic activity, cell development, mRNA processing, and dosage compensation, but their precise mechanisms of action still remain unclear. lncRNAs control gene expression *in trans* or *in cis* by binding chromatin modifiers and transcription factors, and chromatin remodeling affects their repression or activation. The involvement of lncRNAs in modulation of the immune response mechanism has opened up a new era of immune regulation exploration. lncRNAs participate in numerous biological mechanisms of antiviral innate immunity. They are differentially expressed as positive or negative regulators during several important stages of antiviral response, including the expression of virus-induced cytokines, chemokines, and IFNs, the activation of JAK-STAT signaling, host PRR signaling-activated TFs, and ISG transcription. Interestingly, the activity and altered expression of lncRNAs considerably affect the host antiviral immune response mechanism, thereby interrupting viral replication and infection.

Recent studies have provided strong evidence supporting the functions of lncRNAs in the modulation of innate and adaptive herpesvirus immune response mechanisms as well as in host–pathogen interactions. Although several lncRNAs are associated with viral infection, their involvement and experimental validation in herpesvirus infection is very limited. However, research into lncRNA roles in immunity is still in its infancy. This review summarizes the functions of individual lncRNAs in immune responses and lncRNA-mediated regulation of host–pathogen interactions. However, additional studies are required to explore the function, gene expression regulation, and involvement of lncRNAs in viral pathogenesis and immune regulation.

It is worth mentioning particular research topics that should be addressed in the field of immune-regulatory lncRNAs in the future. Functional crosstalk between lncRNAs and miRNAs in the immune regulation of herpesviruses is an important area of interest. Second, defining the key roles of immune-related lncRNAs *in vivo* using animal models will aid understanding of the functions of lncRNAs in the immune response. Finally, lncRNAs are thought to be translated and coded as peptides or small proteins, so it would be beneficial to determine their coding potentials and the roles of coding peptides in immune regulation. A better understanding of the biology of lncRNAs will facilitate our comprehension of their functional mechanisms, and pave the way for the prophylaxis and treatment of many infectious diseases, including herpesvirus infections.

## Author Contributions

Z-FL conceived the research. WA and Z-FL wrote the manuscript. All authors have read and approved the manuscript.

## Conflict of Interest Statement

The authors declare that the research was conducted in the absence of any commercial or financial relationships that could be construed as a potential conflict of interest.
